# Evaluation of Global Experiences in Large-Scale Double-Fortified Salt Programs

**DOI:** 10.1093/jn/nxaa284

**Published:** 2021-02-15

**Authors:** Denish Moorthy, Laura Rowe

**Affiliations:** USAID Advancing Nutrition, John Snow Inc. Research and Training Institute, Arlington, VA, USA; Food Fortification Initiative, Atlanta, GA, USA

**Keywords:** double-fortified salt, fortification, India, effectiveness, feasibility

## Abstract

**Background:**

Double-fortified salt (DFS) is a vehicle for dual fortification with iron and iodine, to reduce their respective deficiencies. This background article is the third in a series reviewing available research, analyses, and experiences on DFS as an effective delivery vehicle for iron and iodine.

**Objectives:**

The objective of this article is to systematically evaluate current programs distributing DFS around the world and catalogue opportunities, risks, and challenges related to programs that incorporate DFS. We carried out a narrative review of DFS programs from around the world with our data sources deriving from a mix of a nonsystematic literature search and interviews with key informants.

**Methods:**

We assessed programmatic experience with DFS from social safety net programs in India (from the states of Bihar, Madhya Pradesh, and Uttar Pradesh) and from non–social safety net country programs or projects in Argentina, Cote d'Ivoire, Kenya, Morocco, Nigeria, Philippines, and Sri Lanka.

**Results:**

Findings revealed color change of the final DFS product was an issue in 9 of the 14 programs or studies reviewed and was the most significant challenge that had a direct impact on consumer acceptance and uptake regardless of type of program (open market or social safety net). Other challenges identified were related to the quality of the salt and lack of DFS formulation standards and regulatory monitoring protocols.

**Conclusions:**

DFS programs need to focus on *1*) improved technology with better consumer acceptance and better performance when used with lower-quality salt; *2*) elucidation and enforcement of DFS formulation quality standards, along with producer incentives; and *3*) strong government backing at the policy level. DFS offers a unique opportunity to leverage an almost universally consumed product with the addition of 2 important nutrients missing in many populations. However, program “maturity” will take time with urgent attention needed for quality production.

## Introduction

Despite the efficacy of double-fortified salt (DFS) in improving hemoglobin, ferritin, anemia, and iron deficiency anemia (1), there has been limited experience with its production and distribution at scale within programs. Across the world, program managers, researchers, and salt production companies have jointly attempted to introduce DFS in various settings and circumstances. However, many of these experiences were/are limited in scope and reach, and most have failed to launch a large-scale DFS program after exploratory discussions. India is the only country that has reported large-scale DFS implementation experience.

Before DFS programs are introduced or scaled up, it is critical to understand where current and past programs have been attempted and/or implemented and assess the factors, whether they be internal, external, environmental, and/or political, that must exist to produce and distribute quality DFS in a scalable and sustainable manner. Larson et al. (1), elsewhere in this supplement, show that DFS affects health outcomes through a program impact pathway that relates to the production, supply, distribution, and consumption of the fortified salt and the biological factors that influence the absorption and utilization of iron in the body. We must also consider factors that can enhance or inhibit the impact of actions along the pathway. The objective of this article is to systematically evaluate proposed, past, and current programs using DFS around the world, and catalogue the opportunities, risks, and challenges these programs faced in incorporating DFS.

## Methods

We reviewed the literature on global DFS programs from peer-reviewed articles, published and unpublished reports, and program documents including a limited number of effectiveness studies that correlated with areas of technical feasibility. An adapted snowballing search strategy was used, using the bibliographies of included articles or reports to identify new relevant studies. An initial list of key informants to interview was developed by the authors after discussions with researchers and experts involved in DFS programs. Interviews with people on this initial list led us to identify additional individuals to interview. Key informants included policy makers, funders, representatives of international organizations, topic experts, researchers, technical assistance providers to salt producers, producers of DFS products, and program managers. Three different informant guides were developed by the authors for program managers of social safety net programs; retailers or salt producers in an open market system; and policy makers, funders, and international organizations. Because of the large-scale experience in India, 1 of the authors (DM) conducted in-person interviews with most of the key informants associated with Indian programs; both authors participated in remote conference calls for the remaining key information from India and other country programs. All interviews were recorded, where possible, for author validation purposes. Information was synthesized using a theoretical framework adapted from the UNICEF Triple A approach (2) to identify gaps in programs and recommend next steps.

### Framework to assess global DFS programs

**FIGURE 1:**
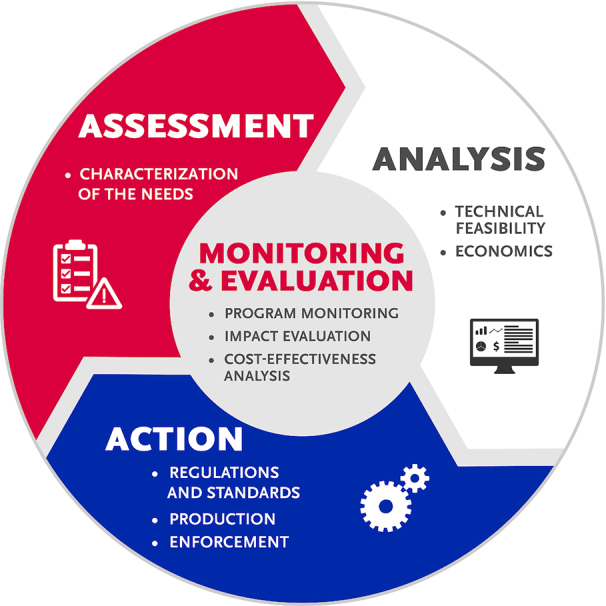
Framework for assessing food fortification programs. Adapted with permission from the UNICEF Triple A approach (2).

We reviewed global DFS programs through an iterative sequence of assessment (characterization of the needs), analysis (of technical feasibility, political will, and economic capacity), and action (enforcement of regulations and standards), supported by a monitoring plan (outputs and outcomes) (**Figure 1**, **Table 1**). We provide results from our interviews in relation to these broad areas.

**TABLE 1:** Description of framework components for assessing food fortification programs^1^

Framework component	Description
Assessment	Characterizing need for the intervention in a particular setting including the availability of reliable data that measure the burden of micronutrient deficiency in the population and the potential effectiveness of various interventions to reduce both inadequacies and deficiencies. Because fortification is one of the many micronutrient delivery interventions available, program managers should have data on the appropriate suite of interventions and understand how the introduction of one will affect the delivery and impact of another.
Analysis	Two elements were reviewed: technical feasibility and economic viability. Within technical feasibility, it is important to ensure characteristics of the fortification vehicle meet criteria to succeed programmatically including whether the food vehicle has wide coverage and consistent consumption; is centrally processed (contains the technology and capacity to meet standards); is compatible with the added nutrients (the addition of micronutrients does not change the appearance, color, taste, smell, and texture of the food and there is minimal micronutrient loss); and is safe to consume. It is also important to assess the comparative economics of fortification programs, especially if different vehicles and distribution mechanisms are being considered for implementation. The respective cost for fortification borne by both the public and private sectors should be explicitly stated.
Action	Focuses on national legislation, regulations, and standards as they relate to DFS formulation and end product quality, packaging, and nutritional claims. Enforcement of standards depends on the existence of external inspections, auditing, and the monitoring regime of the government as well as internal quality control and assurance by the fortifying industries. Programs must provide clear and unambiguous interpretation of the standards and legislation to all stakeholders, transparent testing, and open communication with the private sector (3).
Monitoring and evaluation	Aims to assess the compliance and performance of a fortification program and evaluate its impact on the population in terms of change in biological markers and the risk of excesses. For the purposes of this article, only monitoring is included because, at the time of writing, results of the first DFS program impact evaluation were not available. The evaluation will assess the impact of DFS in the Public Distribution System program in India's Uttar Pradesh state on the reduction of anemia and iron deficiency in women of reproductive age.

^1^DFS, double-fortified salt.

## Results

Using the aforementioned framework, we report findings from 22 interviews conducted between October 2018 and January 2019 (10 over the phone and 12 in-person) and the review of published and unpublished DFS reports and studies. Although the majority of programmatic experience with DFS comes from social safety net programs in India, we attempted to gain insight from other global experiences and open market initiatives. Programs or projects in the following countries or Indian states were assessed: Argentina, India (Bihar), India [Uttar Pradesh (UP)], Kenya, Morocco, Nigeria, Philippines, and Sri Lanka. Argentina and Nigeria were the only countries that had experience selling DFS on the open market. India was the only country that had experience distributing DFS through social safety net programs. Globally, no country required the mandatory fortification of salt with both iron and iodine through retail channels (4).

Of the 8 country programs reviewed, 6 identified color change challenges. The 2 private-sector attempts to sell DFS on the open market and distribution of DFS through India's Public Distribution System (PDS) in some states in India identified challenges with acceptability, cost, and monitoring of DFS quality. Because the Indian DFS program is the largest to date, we provide an overview of the Indian program. Owing to word length limitations, we have added further details on all country programs in **Supplemental File 1**.

### Background on India's DFS program

As a means of addressing India's high prevalence of anemia, in 2011, the Government mandated the use of DFS in 2 social safety net programs: the Integrated Child Development Services (ICDS) scheme and the Mid-Day Meal program (MDM) (5, 6). In 2017, the Government, under the Ministry of Consumer Affairs, Food and Public Distribution, expanded this mandate to include the PDS, a food security system that provides food and nonfood items to households at a subsidized rate (7). The inclusion of DFS in these 3 programs represents the largest implementation of DFS in the world, in terms of number of people reached and total volume of DFS produced and distributed. Social safety net programs were chosen because they reach subpopulations who are at the highest risk of dietary iron deficiency.

### Assessment: characterization of need for DFS

Four areas were considered when assessing the introduction of DFS in any population: proven need, efficacy and effectiveness, contribution to other iron interventions, and co-interventions. We have described these in **Table 2**.

**TABLE 2:** Areas considered under this review when assessing the need to introduce DFS in a population^1^

Area	Description
Proven need	Evidence should exist on the need in the population for an iron intervention to address iron deficiency and iron deficiency anemia. This is important because anemia is often used as a proxy indicator for iron deficiency; however, anemia is a condition with multiple etiologies that are not always addressed by additional dietary iron.
Efficacy and effectiveness	Consideration should be given to the ability of DFS to improve iron intake and reduce iron deficiency and iron deficiency anemia through consistent delivery to, and consumption by, the target population. Larson et al. (1) elsewhere in this supplement cover this topic.
Contribution to other iron interventions	The relative contribution of DFS in comparison with other iron interventions occurring in the target population, such as other vehicles fortified with iron and iron supplementation programs, is important. The Hurrell (8) article in this supplement covers this topic.
Co-interventions	An understanding of the co-interventions that may be in place (e.g., antihelminthic or antimalarial interventions) is needed to accurately interpret the impact of DFS on improving anemia prevalence relative to other anemia-control interventions, and to inform the safety of providing iron in areas with parasitic infections. This topic is not explicitly covered in this supplement because the focus is on fortification programming.

^1^DFS, double-fortified salt.

Findings suggest there have been limited considerations of what proportion of anemia etiology is due to iron deficiency before introducing DFS into a population. Most implementers assume that 50% of the anemia is due to iron deficiency; however, global evidence now suggests this figure is context-specific and may overestimate the percentage of anemia associated with iron deficiency (9). Exceptions to this include India, Sri Lanka, and Bangladesh (although this country was not formally assessed). In India, modeling results showed that the median risk of dietary iron deficiency is 65% (10) (however, given the size and variation of India, the burden of iron deficiency should be considered on a state-by-state basis) and the Government of UP has included an assessment of iron stores in addition to prevalence of anemia in their baseline and follow-up surveys. In Sri Lanka, it was noted that iron deficiency anemia prevalence is 7.3% (11) and other causes of anemia need to be further understood before additional iron interventions are introduced. Finally, although the authors of this report were not able to contact anyone in Bangladesh regarding their DFS experience, the Shields and Ansari (12) article elsewhere in this supplement states that Bangladesh stopped voluntary DFS production in 2011 owing to national data indicating low iron deficiency and iron deficiency anemia. Although other country programs interviewed had an understanding of national prevalence of anemia, they did not have information on the causes of anemia.

Similarly, although efficacy has been proven (1), decision-makers did not consider global evidence of DFS effectiveness when implementing programs. The exception to this is in the Indian state of UP where the state government decided to conduct its own impact evaluation. Results are pending and will drive the state's decision on whether or not to continue the program. The Government of Sri Lanka noted the need to rely on other, existing iron interventions in the country, such as iron supplementation and micronutrient powders, which have achieved high coverage, before introducing DFS. Along the same lines, the Government of Kenya used a precautionary approach and only considered a list of predetermined food vehicles for iron fortification; salt was not on the list. The only explicit consideration of co-interventions among programs was in Kenya where the Ministry of Health feared that adding iron to another food vehicle could increase the prevalence of malaria and its associated mortality.

### Analysis: technical feasibility, acceptability, perceptions, political willingness, and economics of DFS

#### Technical feasibility (organoleptic changes, technical production, and salt quality)

In the programs/projects reviewed, 3 main forms of iron were used in DFS premixes:

1. Encapsulated ferrous fumarate (EFF)—DFS Type 1b and Type 1c
2. Ferrous sulfate—DFS Type 2
3. Micronized ground ferric pyrophosphate—DFS Type 5.

Each form of iron comes with its own set of pros and cons, outlined in other articles (12) and by Hurrell (8) in this supplement. Programs are increasingly using Type 1c as the primary form of iron in the DFS formulation owing to its relatively high bioavailability. In 2015, the Government of India formally approved both the Type 1b/1c and the Type 2 DFS formulations. We found no other national adoption of DFS standards. In general, DFS formulations are intended to provide 100% of one's daily dietary need for iodine and ∼30% of one's daily dietary needs for iron (13) assuming a salt intake of 10 g/d.

##### Organoleptic changes in country programs

Informants mentioned color change of stored salt or of cooked foods in 6 of the 8 programs or studies reviewed, including those in India (4), Argentina, Sri Lanka, and Nigeria. Findings suggest that color change of the stored salt, and in cooked food, was the most significant challenge that had a direct impact on consumer acceptance and uptake. No program reviewed reported change in taste or smell when using DFS. **Table 3** provides a summary of the relation between the form of iron used and the color change observed. A brief description of each country program and further details regarding color change issues can be found in Supplemental File 1. Research studies were included if they provided guidance to countries on the considerations of initiating a DFS program and/or additional context in countries where programs were initiated.

**TABLE 3:** A summary of DFS programs reviewed and the relation between the form of iron used and the color change observed in each program^1^

Country	Type of study, project, or program	Iron form in DFS formulation used	Color change experienced
Argentina (interview)	Open market sales and distribution beginning in 2006 and continuing to date	Type 5	Yes (the DFS turned a slightly brown and yellow color once on the market)
India (Bihar) (interview)	Pilot PDS, ongoing program evaluation	Chelated FS with biopromoters (iron absorption enhancers)	Yes (presence of small black flecks in the DFS after being stored for a period and the metallic spoon used to scoop the salt turned black when left in the salt container)
India (Uttar Pradesh) (interview)	10-district pilot PDS program under evaluation	Type 1c	Yes (cooked food changed to a darker color; also foam occurred in the water used for cooking when DFS was used)
		Salt producers based in Gujarat, selected through a competitive tender process, produced this DFS	
India (Madhya Pradesh) (interview)	Distribution through PDS in 89 tribal blocks in 29 districts	Type 1c	Yes (darkening of food when cooking with DFS and black spots in the salt samples)
Kenya (interview)	Pilot program	Type 1b	No
Nigeria (interview)	Open market sales and distribution. Viable market? No	Type 1b	Yes (DFS changed to a gray and yellow color when stored)
Philippines (e-mail correspondence)	Laboratory research phase to increase shelf life from 3 mo to 2 y	Unspecified	No
Sri Lanka (interview)	Quasi-experimental, randomized controlled trial over a period of 9 mo starting in 2012	Type 1b	Yes, but still acceptable in spite of color change (floating brown particles when boiling potatoes and eggs)

^1^DFS, double-fortified salt; FS, ferrous sulfate; PDS, public distribution system; Type 1b, encapsulated ferrous fumarate using a fluidized bed to agglomerate particles; Type 1c, encapsulated ferrous fumarate using extrusion to agglomerate particles; Type 5, micronized ground ferric pyrophosphate.

##### Technical production: is color change expected?

Key informants involved in the creation of DFS formulations stated that small changes in color are expected during both production of DFS and its use in cooking. **Table 4** outlines explanations of why this color change is expected.

**TABLE 4:** Rationale for color change in the production and use of DFS^1^

Discoloration	Description
During production	Due to the partial denuding of encapsulated compounds by the abrasive mixing process during salt blending, leaving the encapsulated compound partially exposed. The water-soluble iron appears as black spots among the white salt grains.
During DFS use in cooking	Results from breaking down of iron encapsulation, which allows iron to be released into the food, subsequently causing foods to turn a darker, sometimes reddish color.
Nutritional implications	The interaction between *encapsulated* iron in DFS and iodine is limited. According to key informants, studies conducted at the University of Toronto indicate that any detrimental interaction of encapsulated iron with iodine would take weeks to occur and, when it did occur, would result in a loss of only 1%–2% of iodine. Losses with *unencapsulated* iron sources, however, can be ≤80% over a 2-mo period.

^1^Key informants involved in the creation of DFS formulations stated that small changes in color are expected during both production of DFS and its use in cooking. The table outlines explanations of why this color change is expected. DFS, double-fortified salt.

##### Quality of salt: purity, particle size, and moisture content

Current DFS specifications in India require 98%–99% purity of salt, which limits the number of DFS producers (14). Indian consumers, in particular, prefer coarse salt (which implies higher moisture content, larger particle size, and lower purity). A high moisture content and a large particle size mean greater loss of iodine in the DFS and/or a rapid color change to dark yellow, regardless of the form of iron used. Therefore, there is a need to address the threshold whereby moisture content and particle size cause a decrease in iodine and a change in color. Larson et al. (1) elsewhere in this supplement present results that point to loss of iodine as a result of salt quality and/or DFS formulation quality, whereas other results do not show such losses. Greater clarity is, therefore, needed around what may cause loss of iodine in a DFS product.

#### Acceptability and perceptions

Color change was a significant overarching factor when it came to consumer acceptance, however, there were also other factors that created misperceptions.

DFS in India is a commodity supplied only through social safety net programs. When it is received through the PDS, it is a part of a food basket bundle, which is a predetermined basket of commodities (both commodities and amounts are predetermined) that PDS enrollees receive, regardless of their personal preference, in fair price shops, which are shops that distribute PDS baskets of food and other goods. Several of those interviewed indicated that DFS was included in the food basket bundle because there would not be enough demand, by shop owners or consumers, to purchase the product otherwise. In this case, there is no choice given to the shop owner or the consumer when it comes to purchasing DFS, leading to impressions that the consumer and the shop owner are being forced to buy DFS. This top-down approach, coupled with color change, may feed the perception that fair price shop goods are of inferior quality. One key informant reported that PDS beneficiaries expressed that the quantity of DFS was “too much” and that excess DFS was being pushed through to households; if there are supply delays, then they get double their monthly allotment (≤4 kg/mo). For commodities like rice and wheat, excess quantities supplied to households are informally resold in the retail market; however, there is no unofficial market to absorb extra DFS and many households end up storing the excess salt. Owing to perceptions of inferior quality due to color change, which could be exacerbated by extended periods of poor-quality storage at the household, the beneficiaries discard it.

In the UP program, fair price shop owners are expected to communicate the importance of DFS to their customers. However, in reality, there is often very little time for detailed interaction with consumers.

In Argentina, the salt producer attempted to roll out a TV campaign to increase DFS sales; however, the government saw the campaign as a means of counteracting their ongoing salt reduction strategies for noncommunicable disease (NCD) prevention. As a result, the salt producer discontinued the campaign. Similarly, the private salt producer in Nigeria attempted a radio program to address color changes; however, these efforts did not produce any meaningful change in purchase or use patterns of the DFS.

The notion of *how* the DFS product is marketed to consumers was also raised. After the salt producer in Argentina released their DFS product, a local competitor released a multifortified salt, which was fortified with iron, folic acid, vitamin C, and magnesium. The product did not succeed, it was explained, because the addition of so many nutrients turned the perception of salt into that of a medicine. Although marketing DFS is not necessary when fortification is mandatory, it may lead to negative perceptions about the product and uptake challenges.

#### Political willingness

The introduction of DFS as a public health intervention requires the support of government to ensure it is a product that is accepted and/or endorsed at the policy level and to ensure it is monitored for quality purposes. In general, our findings point to much variability in government support for and commitment to DFS efforts. In India, political support was seen at the national level; however, it was mixed at the state level. At the national level in India, support has been demonstrated through a mandatory policy that requires the use of DFS in the MDM and ICDS, which use salt for cooking; however, use is only mandatory through the PDS if salt is already provided by that state. Support has also been demonstrated through the committed work of the Food Safety and Standards Authority of India (FSSAI) around the adoption of standards and monitoring protocols, although the enforcement of these protocols by the states is unknown. In 2018, India's Prime Minister Narendra Modi gave an address on the problem of anemia, its negative effect on national gross domestic product, and the imperative role that specifically DFS could play in prevention. At the state level, however, political commitment was equivocal because of the financial obligations on the state that DFS adoption would require.

Kenya and Sri Lanka demonstrated little government support at national or state levels. Kenya's Ministry of Health perceived an increased risk of malaria with the introduction of DFS. In India, Sri Lanka, and Argentina there were perceived conflicts of interest at the government level between DFS and national NCD prevention programs [e.g., salt reduction efforts as was the case in Sri Lanka (15)].

##### Providing DFS through India's PDS: opportunities and challenges

Although distribution of DFS through India's social safety net programs provides an effective means of reaching a nutritionally vulnerable population, there remains a difficult balance that must be achieved between the national mandate for DFS and state-level autonomy. In 2017, the Government of India made it a requirement to supply DFS in the PDS *if* salt is a commodity provided in that state's PDS program. Because salt is not universally provided across state PDS programs, the decision to include DFS is at the discretion of the state government.

Motivating state governments to include DFS in their social safety net programs is dependent on a number of factors, including political, financial, and administrative incentives. In addition, state decision-makers are inherently risk adverse, and usually require clearance from the Department of Health to vouch for the efficacy and safety of an intervention. The process of DFS procurement within the PDS and the steps where it can break down are described in the text and Supplemental Box 1 of Supplemental File 1.

#### Economics

Key informants had differing opinions on whether price was an issue in the uptake of DFS for governments, producers, and consumers.

##### Cost to producers

Producers in Nigeria and Argentina did not find the production and open-market sale of commercial DFS a cost-effective business model. In the case of Argentina, the DFS product is priced at 40% more than wholesale iodized salt and sold as a premium product targeting higher-end consumers. Producers in Nigeria also thought that demand for the product might trickle down to bottom-of-the-pyramid consumers. However, after initial DFS commercialization, companies selling salt in the retail market agreed the DFS product was not reaching bottom-of-the-pyramid consumers and sales, across the board, were poor. In Argentina, for every 100 packets of iodized salt sold, 5 packets of DFS are sold; however, information on actual population coverage of DFS in Argentina was not available. Depending on the formulation used, the cost to procure the DFS formulation for the Argentinean producer ranged from USD $24 to $31/ton compared with USD $0.78/ton for iodine, warranting a significantly higher price for their DFS product than for the same company's iodized salt product.

##### Cost to government

The cost to government to provide DFS through India's social safety net program is significant given the amount of subsidy provided. The UP Government, for example, provides an INR 6–9/kg subsidy for DFS (it is procured for INR 12/kg and is provided for INR 3/kg or INR 6/kg depending on the type of beneficiaries). Assuming an 11-g/d intake of salt (16), an average consumption of 4 kg/person-year, and a target coverage of 24 million beneficiaries, a DFS subsidy for the Government of UP ranges from INR 576 million to 1.1 billion (USD $8 million–$15.5 million) for 1 y for 10 districts (which is only a subset of the state; UP has a total of 75 districts). Similarly, the Government of Madhya Pradesh (MP) procures DFS at INR 8/kg and sells at INR 1/kg, for a subsidy of INR 7/kg. With a targeted coverage of 12 million beneficiaries in MP, the DFS subsidy for the Government of MP is INR 336 million (USD $5 million) for 20 districts. A scale-up of DFS to all the districts in UP (75) and MP (52) would raise the query of whether such costs are sustainable (particularly at the state level) over time.

##### Cost to consumers

In order to make a profit, producers stated the need to price DFS at 30%–50% above the price of iodized salt. The private salt producer in Argentina found the price of their DFS to be inversely correlated with purchasing patterns. However, India's UP program stated that price did not play a large role; instead, it was a matter of preference for different types of salt. Others noted that price was not an issue as the spending per family on salt is, relatively speaking, low. In the Abdul Latif Jameel Poverty Action Lab (J-PAL) program in Bihar, a pricing experiment was conducted that provided DFS coupons to consumers at different price points. They found that demand fell sharply at a price of INR 10/kg, the price of the cheapest alternative branded salt, with under one-third of households willing to purchase just below INR 10/kg. When discounted further to INR 9/kg (from the retail price of INR 20/kg), only ∼20% of households purchased the DFS; this dropped to 10% after 3 y. Shop owners were not buying large quantities of the product because of the low profit margin even with the discount they were provided. However, price may not have been the only factor. In this program, DFS was only available in larger packages, whereas iodized salt was available in multiple sizes, including small packages more attractive to consumers with less money (17).

### Action: regulations, standards, and monitoring of DFS

#### Quality standards and regulatory monitoring

Based on this report's findings, one of the fundamental challenges faced globally in the production and rollout of DFS is the lack of DFS formulation standards and regulatory monitoring protocols. Decades of experience implementing national fortification programs have demonstrated the importance of ensuring that formulations and fortified products meet quality standards. Not doing so risks precluding any sort of positive nutritional impact.

#### National DFS standards and ability to enforce

As noted earlier, in 2014 the Government of India adopted and approved a DFS national standard using EFF; in 2015, the Type 2 DFS formulation was added to this approval. In theory, FSSAI food safety officers (government personnel) are responsible for monitoring DFS in MDM and ICDS; in UP, staff from Tata Trust assist with monitoring the supply of quality DFS through the PDS. This includes ensuring a monthly supply to fair price shops, routine checks on households that are supposed to receive it, and sampling of salt for quarterly quality tests conducted by FSSAI. This monitoring does not take place at the point of production. The reliability and sustainability of the monitoring, by both FSSAI and Tata Trust, or the ability of Tata Trust to monitor additional states, are not known. The actual testing protocol for DFS is currently under review by FSSAI. A test kit to detect fake samples is also being explored. Long-term, Tata Trust plans to train government staff on how to monitor DFS but currently they are doing all the on-the-ground work to ensure quality. Tata's investment for monitoring in the State of UP alone is USD $2 million. Will government have the will, the financial ability, and/or the technical capacity to take on these efforts in the future, particularly given the vulnerability of state governments to budget cuts? How can oversight increase around prequalifying suppliers as a means of bringing down traditional regulatory monitoring costs and checking that these are the only suppliers used as sources?

#### Global and/or national DFS formulation standards and ability to enforce

Interviews, largely related to India's programs, voiced the need to manage quality control at the formulation level. At the time of writing, a *standard for DFS formulations* does not exist. This is significant because it means there is no guidance around what the DFS formulation should look like, and particularly important because, without proper encapsulation, coating, and masking agents, premixes risk exposure of iodine to oxidizing iron, which in turn cause color change and potentially loss of iodine. The University of Toronto is exploring ways to develop a proposed global DFS formulation standard, with methodologies for testing the quality of the encapsulation and titanium oxide coating. However, equipment needed to test the iron coating will likely not be available in settings where testing capacity is limited, necessitating finding another means to determine the quality of DFS formulation or improved testing methods that can be realistically implemented.

## Discussion

DFS offers a unique opportunity to leverage an almost universally consumed product with the addition of 2 important nutrients missing in many populations. However, more work needs to be done and program “maturity” will take time depending on key areas, outlined below, that still need urgent attention.

Based on the findings, stronger efforts are needed around 7 specific areas, as follows.

### Need

Greater consideration is warranted at national decision-making levels around the need and safety of DFS interventions. Of the countries reviewed, Sri Lanka and Kenya showed 2 facets of need and safety, where low iron deficiency from micronutrient status assessments (Sri Lanka) and fear of increasing adverse events (Kenya) led to abandonment of DFS plans. In countries where other iron delivery interventions already exist, introduction of DFS will need to consider whether it is complementary to or in place of existing interventions (Sri Lanka).

### Technology

There is a need to identify the root causes of color changes in DFS, because these will inform how they can be addressed or communicated. If there is more work to be done technologically (e.g., more appropriate microencapsulation of the iron), this must be carefully balanced with DFS production costs and with what we can expect for the uptake of a product that changes the color of food.

### Product expectations

If color change cannot be avoided with the current technology, the question of how to manage a product produced at scale with organoleptic changes will need to be answered. Currently, as a result of these color changes, consumers do not demand the salt and assume it should not be eaten, producers do not have a market for it, and social safety net programs risk significant leakages outside of the household and/or limited uptake. Communication strategies may need to be put in place so there are clear expectations among producers and consumers regarding the product.

### Quality monitoring

Four factors are key to ensure the quality production of DFS: *1*) a national and/or global standard that stipulates the quality of the iron compound used in the DFS (this does not exist globally), *2*) a national standard that indicates the amounts of iodine and iron that are required in the DFS product (India has this in place), *3*) a global or national standard that stipulates the quality of salt needed to produce DFS (India has this in place), and *4*) the in-country ability to test, enforce, and/or otherwise ensure a quality DFS formulation and end product (this is limited globally). National standards should also include salt purity content, particle/grain size, and moisture content given the impact of salt characteristics on DFS quality. However, the ability of salt producers to adhere to these standards needs to be considered. Without strong enforcement, the program's nutritional impact is jeopardized but it also removes any incentive for the private sector to produce a quality product, particularly when the open market forces of supply and demand are not at work.

### Role of government

Government support is critical for *1*) adoption and dissemination of a quality standard for the DFS formulation and the end DFS product; *2*) regulatory monitoring and enforcement to ensure quality standards are met; *3*) clear messaging around procurement of quality DFS; *4*) clear messaging around benefits of DFS; *5*) ensuring complementarity with other iron interventions; and *6*) a clear procurement process and list of preapproved producers of DFS formulations with the ability to enforce the standards at the state/national level. Conducting a political mapping or landscape analysis before initiating DFS work in a country should be considered to allow for a better understanding of constraints and perceptions that may exist within the national political environment and to enable attempts to address such perceptions.

### Cost

Several questions related to cost still need to be answered. What is the breaking point for the sale of salt on the open market and can DFS realistically be sold for that price? In terms of subsidies, can state governments sustain costs in the long term? If not, what can be done to bring costs down technologically and/or via consumer demand?

### Open market testing

In India, large-scale table salt companies are interested in selling DFS in the retail market, even though they are hesitant to enter the market right away. The top 5 salt companies control 40% of the salt market; the top 10 salt companies control 60% of the market. Each of these companies has moved DFS development to their R&D department where their formulations are being subjected to lengthy and rigorous internal quality testing. This will take time but the results will be informative for next steps.

By using India's social safety net programs as the testing ground for DFS, the DFS program may have missed an opportunity to develop an effective open market product like iodized salt. The only way thus far to guarantee any market for DFS at this time is through the social safety nets. In order to prove product viability, producers need to be accountable to the end consumer; the product needs to be subjected to consumer preference or open market competition. Producers have no real accountability to the end consumer currently because the end consumer does not choose, in most cases, to purchase the product. Therefore, little is known about what would increase sales, acceptability, and ultimately consumption. What we can learn from the open market may be the missing link needed to understand true effectiveness and to move this intervention out of its infancy stage and into a stage that would demand greater uptake. At the same time, we do not want to lose sight of the momentum that has been built around this innovative product.

### Conclusions

The authors recognize these findings are not representative of all DFS experiences to date. Results are intended to guide discussions and inform related questions regarding the current status of DFS implementation. Gaps in this research include greater insight into India's state-level DFS procurement process, specific factors that lead states to incorporate DFS into their social safety net programs, clarity around how DFS will be funded long-term in India on current state budgets, and how DFS products will be monitored in India without outside assistance.

Given the effectiveness gaps outlined in this article, there is a need to carefully reflect on the challenges that currently exist with DFS products, how these challenges can be addressed *before* further rollout occurs, and how, as program implementers and decision makers, we can match a true nutritional need for DFS with a product that the consumer will accept. Without those steps, we risk jeopardizing the current and future success of this commodity.

## Supplementary Material

nxaa284_Supplemental_File

## Notes

Supported by United States Agency for International Development (USAID) contract 7200AA18C00070 awarded to John Snow Inc. Research & Training Institute (to DM) and the Food Fortification Initiative (to LR).

Author disclosures: The authors report no conflicts of interest.

Published in a supplement to *The Journal of Nutrition*. The Guest Editor and Supplement Coordinator for this supplement publication was Becky Tsang, Iodine Global Network, Seattle, WA, USA and she reports no conflicts of interest. Publication costs for this supplement were defrayed in part by the payment of page charges by the Iodine Global Network. The opinions expressed in this publication are those of the authors and are not attributable to the sponsors or the publisher, Editor, or Editorial Board of *The Journal of Nutrition*.

Abbreviations used: DFS, double-fortified salt; EFF, encapsulated ferrous fumarate; FSSAI, Food Safety and Standards Authority of India; ICDS, Integrated Child Development Services; MDM, Mid-Day Meal; MP, Madhya Pradesh; NCD, noncommunicable disease; PDS, Public Distribution System; UP, Uttar Pradesh.
