# Supplementary material for: Evaluation of Global Experiences in Large-Scale Double-Fortified Salt Programs
Source: J Nutr. 2021 Feb 15;151(Suppl 1):38S–46S. doi: 10.1093/jn/nxaa284 (PMC7882358; doi:10.1093/jn/nxaa284)
Supplement: nxaa284_Supplemental_File [file nxaa284_supplemental_file.docx]

**Evaluation of Global Experiences in Large-Scale Double Fortified Salt (DFS) Programs**

**Denish Moorthy**

**Online Supplementary Material**

**Background on Country Programs**

*India*

Since 2016, the Government of UP, assisted by a non-governmental agency, has been involved in a 10-district pilot program to assess the impact of distributing DFS through the PDS to address micronutrient malnutrition. The program ultimately aims to reach 24 million beneficiaries. Although the program was temporarily suspended in 2017 due to a change in government, it resumed in 2018. Its effectiveness is being evaluated to inform the government’s decision on whether to scale-up DFS distribution in the PDS program; study results are expected to be made public by end of 2019. This pilot program involves, among other things, selling DFS at various subsidized prices to PDS recipients. Multiple reports from the UP pilot have indicated that when DFS is used to cook food, the food changes to a darker color. In addition to color changes, the presence of foam in water when using DFS was reported. The presence of foam appears to be due to the breakdown of the coating around the encapsulated iron, causing a soapy agent to form. This caused a media backlash in UP, which affected consumer uptake of the product.

In MP, the state government is providing DFS through PDS shops to 89 tribal blocks in 20 districts, with a target of reaching 12 million beneficiaries in the districts. The procurement of DFS is conducted by the state, and implementation is supported by a non-governmental agency. The non-governmental agency assists with monitoring indicators, behavior change, and demand creation around DFS, logistic and supply chain management, training of fair price shop owners, inspection of warehouses where DFS is stored, quality assurance and control, evaluation of the program, and stability studies. In this program, there have been reports of darkening of food when cooking with DFS and black spots in the salt samples; to address this, the program managers have included consumer education regarding the color change as part of their demand creation and behavior change strategy.

Similarly, a study lead by MIT’s Abdul Latif Jameel Poverty Action Lab (JPAL) in the state of Bihar looked at the potential impact of a subsidy on DFS to assess consumer demand and uptake, the effect of marketing approaches, and nutritional impact. Their pricing experiment reported the presence of small black flecks in the DFS after being stored for a period. Additionally, the small metallic spoon used in most households to scoop the salt out would turn black when left in the salt container. Because of this, consumers assumed the salt was no longer fit for human consumption and instead fed the salt to their animals. In this study, despite the fact that DFS was provided for free in a subsample of the randomized villages to understand efficacy, uptake was the major issue due to leakage of the salt out of households to animals. Efforts were made to counteract the lack of acceptability by the end consumer by creating a movie that talked about anemia and DFS and the observed color change in the context of a classic Hindi film. This marketing approach did have an effect on increasing the uptake of the salt but it was limited. Uptake of DFS throughout this study was 10-11 percent and increased only slightly with the communication strategy.

The color change seen in the UP, MP, and Bihar programs is consistent with findings of a study conducted in southern India by Andersson et al which found that when DFS, made with EFF as the source of iron, was added to the water used when cooking rice, it produced small black spots on the surface of the cooked rice grains (1). Color change was not found when micronized ground ferric pyrophosphate was used in place of EFF in this study (1)

**Providing DFS through India’s PDS: Procurement Process**

The trajectory of DFS in India is intended to scale up from targeted public programs to retail market in order to target the whole population (2). It is important to note that procurement within PDS programs is dependent on money raised and spent by the different states of the country because health, and by extension nutrition, are subjects under the jurisdiction of the state governments in India. (3)

**Supplementary Box 1** outlines the steps that a state government takes to procure DFS for its PDS. In our review, we hypothesize a breakdown between Step 2 (identifying DFS producer) and step 6 (monitoring DFS quality). There is a lack of clarity around the DFS procurement process for PDS at the state-level. The opaque and poorly enforced procurement requirements in India, noted by several individuals interviewed, is one potential reason for consistent color change issues and poor quality DFS on the market. As one individual noted, “the organoleptic [issue] is out of their [the program implementer’s] hands because there is no control over the source of DFS.” This statement assumes a program implementer is not the government. Another noted that the “procurement is murky and big companies do not bid on these tenders because of the murkiness.” Tenders may be awarded to salt traders or producers who might procure poorly encapsulated premix for their salt, leading to dark spots of iron visible in the salt. Finally, another individual noted, “they [premix suppliers] are cutting costs and not coating the premix, which causes color change. They are cutting costs by reducing titanium dioxide coating on the [encapsulated] ferrous fumarate.”

The potential breakdown in step 6 (monitoring DFS quality) has an impact on DFS quality. Without strong oversight into where DFS is being sourced from and how its quality is being ensured, it becomes easy for producers to make adjustments in areas where it could benefit them the most economically (i.e. premix encapsulation and coating). Without encapsulated iron and/or without a titanium dioxide coating on the EFF, color change in the form of dark spots or flecks will occur.

1. A public tender is released. (Except in TN where the state government has set up a DFS production facility to supply the state’s PDS requirements; therefore, no public tender is released).
2. A producer is identified. These producers are to be certified producers that have applied for approval by India’s Food Fortification Resource Center (FFRC), which operates under the Food Safety and Standards Authority of India (FFSAI), the national food regulatory body. The approval process includes the submission of samples to FFRC and FFRC confirmation that samples comply with national standards. State governments should be aware which suppliers have been pre-approved and which have not. However, the extent to which this process is followed is unclear.
3. Salt is produced, purchased, and transported to a state-government warehouse. Since procurement happens at the state-government level, only the state government has explicit control over where salt is procured. The state government takes on the responsibility (and the cost) of procuring any commodity that is to be distributed through the social safety net program. In practice, however, the government may or may not choose to source from a supplier that is known to produce high quality salt or procure premix from high quality suppliers. Outside entities are not allowed to interfere in the tendering or procurement process or between production of the salt and delivery to a state-government warehouse.
4. Fair price shop owners are then required to purchase bundled “food baskets” (e.g. rice, wheat, and salt) from the state-government warehouse, which, depending on the state, include DFS. In practice, not all states include salt; thus not all states include DFS in their PDS food basket. The decision regarding what to provide and what to bundle is made on a state-by-state basis. The consumer, who then purchases the bundled “food basket” from the fair price shops, automatically receives what is in the bundle (e.g. DFS, alongside rations of wheat grain, rice, oil, and kerosene, if applicable).
5. For the UP program, Tata Indian Nutrition Initiative (TINI), a project of Tata Trust, worked to ensure the uninterrupted supply of DFS from the warehouses to the fair price shops each month, in addition to routine household checks to ensure purchase, supply, and quality. The ability of Tata Trust to monitor additional states, is not known. Currently, Tata Trust is only monitoring in the state of UP.
6. However, in lieu of TINI, FFSAI food safety officers are responsible for checking DFS quality within the PDS, MDM, and ICDC programs.

**Supplementary Box 1: State government process for procuring DFS in the PDS**

However, there is currently a gap in knowledge regarding when and why color changes occur when EFF is used as a fortificant. The cause of dark spots in EFF-fortified DFS is attributed to several issues, including poor encapsulation and/or titanium coating, iron interactions with less-refined (e.g. higher moisture) salt, or other inadequate production practices.

Further complicating the challenges of procuring and monitoring DFS, is the existence of logistic and programmatic issues within the PDS system that affect DFS implementation under this platform. Several studies point to corruption, albeit decreasing (5), often in the form of leakages (5, Overbeck 2016) within the PDS over the years. PDS “leakages” refer to the proportion of commodities released by the Food Corporation of India (FCI) that fail to reach consumers (5). Other forms of corruption may also exist.

Uniquely in India, a large subsidy program exists that demands significant amounts of state funding, no national or global measures in place to define high-quality premix, limited national measures in place to ensure end product quality, and a public social safety net system where historically, under-the-table transactions have reduced program effectiveness. In attempts to understand the procurement process, the authors found it particularly difficult to obtain any information from any key informant on this topic. Concerned individuals were not inclined to talk about the matter. As a result, the authors believe there is likely a relationship between the lack of clarity around DFS procurement at the state level, inherent social safety net challenges, DFS quality issues, and the production and procurement of DFS as a viable product that is acceptable to consumers.

*Argentina*

In 2006, a private salt producer in Argentina released a DFS product on the open market using micronized ground ferric pyrophosphate as the form of iron with the goal of producing a product that could help improve the nutritional status of the population. This private producer is one of two companies globally with experience selling a DFS product on the open market (the second company is based in Nigeria), and is the only company globally still selling DFS on the open market. This producer noted that the DFS turned a slightly brown and yellow color once on the market. The salt producer conducted a series of focus groups prior to the launch in 2003 that revealed consumers increased trust in a product that had a different color. According to the focus group findings, in the absence of a color change, there was a belief that salt producers were lying about the nutritional value of the product. However, the company believes this perception may have changed today. The producer continues to produce DFS for the retail market. Prior to using micronized ground ferric pyrophosphate, the producer used a premix with FS and experienced similar color change issues.

*Nigeria*

A second company, based in Nigeria, has experience selling a DFS product on the open market. In 2001, they released a DFS product in a small area of eastern Nigeria using EFF. The premix and blenders were provided by UNICEF and the salt was sold at a premium (price differential data were not available). Nigeria was thought to be a promising location to test DFS on the open market due to its relatively consolidated salt industry and the universal consumption of refined salt. However, sales were extremely low due to what the salt producer described as a “foundational color issue” as the salt changed to a grey and yellow color when stored. In order to address this challenge, the salt producer launched a radio program to inform consumers but it had little effect. As a result, the private sector in Nigeria did not feel that consumers would be willing to use the product even under a mandatory fortification program. Further efforts were discontinued in Nigeria and the company no longer produces their DFS product.

Color change issues, albeit in cooked food rather than in the stored salt itself, were also reported in a study conducted in Nigeria in 2003. Using microencapsulated iodine (instead of microencapsulated iron) with ferrous fumarate, a marked darker color was seen in green vegetables cooked with DFS. Despite this finding, participants reported DFS to have better flavor, sharper/saltier taste, to be whiter and grainier, and flow better compared to iodized salt (4).

*Sri Lanka*

In Sri Lanka, a quasi-experimental, randomized controlled trial was carried out in 2012 at the community level to assess the effectiveness of DFS intake on hemoglobin and ferritin concentrations in school-aged children (boys and girls 5 to 10 years of age). Changes in color of the salt were reported by 5.7 percent of the participants in the DFS group compared to 2.7 percent in the iodine group. Color change included the occurrence of floating brown particles when boiling potatoes and eggs. This was reported as the main problem in the acceptability, willingness to purchase, and utilization of DFS in this study. However, actual rates of acceptability remained high as measured by a response rate of 95.3 percent reporting an interest to continue using the salt (5). Although this was a research study, the findings were used to inform programmatic decisions.  Sri Lanka conducted this study because there was interest in evaluating a new option for the delivery of iron. Because iron deficiency was not found to be a problem nationally, however, the decision was made not to move forward with the introduction of DFS.

*Kenya*

Organoleptic issues in Kenya were not reported in interviews on early DFS efforts in the country. However, color and texture changes were reported in an unpublished thesis/dissertation from a candidate at the Applied Nutrition Program at the University of Nairobi. Despite the fact that the color and texture of DFS was preferred less compared to iodized salt (some participants found DFS to look dirty or have dark particles), the study reported DFS to be acceptable for use among participants in all sites (6).

Outside of these smaller studies, the Canadian International Development Agency (CIDA) targeted Kenya in 2004 to pilot DFS on the open market because of its consolidated salt industry and universal consumption of refined salt (similar to Nigeria). After organoleptic tests, efficacy trials, and focus group discussions around price points, industry was eager and ready to launch a commercial DFS product as a new brand of salt alongside their already well-established brands. They wanted to launch first as a premium product as an easy entry point and then expand to lower pyramid purchasers. In the end, the project faced resistance from the Ministry of Health, who wanted large-scale efficacy trials conducted in light of fears that adding iron to another staple food would lead to increased prevalence of malaria and associated mortality.

*Philippines*

A salt company in Philippines has been carrying out market and laboratory research on DFS for the past four years. The key informant reported that the shelf life of the product being developed is 2-3 months, after which time there is a loss of iodine in salt because of the added iron (percentage of iodine lost was not reported). They are currently developing the product further with a target of a two-year shelf life, as per the Philippine Food and Drug Administration standards for iodized salt. They have not brought a DFS product to market but their aim is to market the salt in the health sector space, with a focus on pregnant women and people diagnosed with iron deficiency.

*Morocco*

In a series of studies conducted in northern Morocco, findings indicated color change had a direct relationship to the moisture content of the salt (6). No significant color change was found in the DFS in these studies compared to the iodized salt during the dry season when moisture content of the salt is low (<1 percent). Even after 20 weeks of storage, there was no visual color change. However, when the moisture content of the salt increased during the damp season to about 3 percent, the DFS developed a mild yellow color during storage and there was a significant difference in the color of the salts at 8, 12, and 20 weeks. Seventeen percent of individuals reported that the salt changed the color of foods; 15 percent reported cooked sauce with milk and onion turned a pale gray color; and 2 percent reported a pale gray color in salads that contained onions (6).

**Supplementary References**

1. Andersson M, Thankachan P, Muthayya S, Goud RB, Kurpad AV, Hurrell RF, Zimmermann MB. Dual fortification of salt with iodine and iron: a randomized, double-blind, controlled trial of micronized ferric pyrophosphate and encapsulated ferrous fumarate in southern India. Am J Clin Nutr. 2008;88:1378–87.

2. Raman J, Mannar V. Double Fortified Salt in India: Coverage, Efficacy and Way Forward. Indian J Comm Health. 2018;30 Suppl:63–71.

3. Glassman A, Mukherjee A. Getting centre-state relations right for health in India [Internet]. Ideas For India. 2015 [cited 2019 Mar 1]. Available from: http://www.ideasforindia.in/topics/trade/getting-centre-state-relations-right-for-health-in-india.html

4. Keshinro OO, Akinyele IO, Ajayi OA, Sanusi RA, Fadupin GT. A family acceptability study for double fortified salt (DFS) using rural households in Nigeria. Ottawa, Canada: Micronutrient Initiative; 2003.

5. Jayatissa R. Effectiveness of Salt Fortified with Iron and Iodine to Improve Iron status in a Community Settings in Sri Lanka. Colombo, Sri Lanka; 2012.

6. Zimmermann MB, Zeder C, Chaouki N, Saad A, Torresani T, Hurrell RF. Dual fortification of salt with iodine and microencapsulated iron: a randomized, double-blind, controlled trial in Moroccan schoolchildren. Am J Clin Nutr. 2003;77:425–32.
